# Comparison of hepatic PDFF, T2*, and T2 estimates from breath-hold and free-breathing respiratory-gated MRI acquisitions in children and young adults

**DOI:** 10.1007/s00261-025-05232-z

**Published:** 2025-10-15

**Authors:** Anandh Kilpattu Ramaniharan, Justine Kemp, Murat Kocaoglu, Andrew Trout, Jonathan Dillman, Mary Kate Manhard, Amol Pednekar

**Affiliations:** 1 Cincinnati Children’s Hospital Medical Center, Cincinnati, USA; 2 University of Cincinnati, Cincinnati, USA

**Keywords:** Liver, Quantitative imaging, Relaxometry, Pediatric, MRI, Free breathing

## Abstract

**Purpose:**

Respiratory motion during data acquisition may compromise image quality and quantitative accuracy of MRI, particularly in patients with limited breath-holding capability. This study aimed to compare hepatic proton density fat fraction (PDFF), T2*, and T2 estimates of the liver obtained from breath-hold (BH) acquisitions with those from corresponding free-breathing respiratory-gated (RG) MRI acquisitions.

**Methods:**

In this IRB-approved study, healthy controls and patients with liver disease underwent quantitative liver MRI. Hepatic PDFF and T2* were estimated using a confounder-corrected chemical shift-encoded (mDIXON Quant) sequence, and T2 was estimated using a multi-echo Gradient and Spin Echo (mGRASE) sequence with fat suppression, each performed as both BH and RG acquisitions with identical acquisition parameters. Three observers independently drew regions of interest (ROIs) on PDFF, T2*, and T2 maps and agreement between mean values was compared using intraclass correlation coefficients (ICC) and Bland-Altman analysis.

**Results:**

Nineteen participants (15.4 ± 5.5 years; 9 males) were evaluated. Scan times were 7 s and 14 s (BH) and 21 ± 6 s and 28 ± 4 s (RG) for mDIXON Quant and mGRASE respectively. Estimated values from BH acquisitions were: PDFF – median 5% (range 1–43%), T2* – mean 27 ms (range 9–41 ms), and T2 – mean 48 ms (range 34–63 ms). RG and BH acquisitions yielded comparable values with biases of −0.33% [−1.42 to 0.76%] for PDFF, −1.58 ms [−9.50 to 6.33 ms] for T2*, and 0.16 ms [−2.43 to 2.12 ms] for T2, and strong correlations (ICC = 0.99 [0.99, 0.99], 0.85 [0.66, 0.94], and 0.99 [0.97, 0.99] for PDFF, T2*, and T2).

**Conclusion:**

RG acquisitions provide hepatic PDFF, T2*, and T2 estimates comparable to BH acquisitions without substantial time penalty and are a viable alternative for patients with limited breath-holding ability.

## Introduction

Hepatic quantitative MRI provides essential information for clinical decision-making, enabling non-invasive diagnosis, staging, and monitoring of liver disease progression and treatment response [1]. Accurate estimation of hepatic fat content, iron deposition, and shear stiffness is integral to current clinical practice [2, 3]. Proton density fat fraction (PDFF) quantification, which measures the lipid content in the liver, plays a critical role in diagnosing, staging, and monitoring steatotic liver disease [4]. Hepatic T2* estimation, which gives an estimate of iron content in the liver, helps monitor iron-related liver diseases and guide treatment [5, 6], while T2 estimates can be useful in evaluating liver steatosis [7] and fibrosis [8, 9].

While hepatic quantitative MRI offers valuable insights into alterations in tissue properties, its accuracy can be compromised by respiratory motion induced artifacts. Currently, obtaining accurate metrics requires patients to perform breath-holds to mitigate these artifacts, which may compromise the accuracy of estimated values [10]. Spatial resolution and coverage of quantitative hepatic maps are thus constrained by the patient’s ability to suspend respiration. Sustained breath holds are particularly challenging for patients with limited lung capacity or reduced lung function due to comorbidities [11, 12]. Poor breath-holding may necessitate repeated scans, prolonging the overall scan session [13]. Children often struggle with breath-hold instructions and may require sedation to lie still in the scanner and avoid bulk motion artifacts, further restricting the ability to perform breath-holds. Free-breathing alternatives to breath-hold hepatic quantitative MRI can help improve clinical workflow, enhance patient comfort, and minimize the need for sedation.

We hypothesized that synchronizing quantitative MRI sequence data acquisition with the patient’s respiratory cycle could adequately reduce motion induced artifacts, providing adequate accuracy of estimated quantitative MRI metrics. We aimed to compare the hepatic PDFF, T2*, and T2 estimates obtained using breath-hold and free breathing respiratory-gated acquisitions in healthy participants and participants with liver disease, using identical imaging parameters and spatial coverage for both scans.

We utilized previously validated imaging sequences. The six-echo confounder corrected chemical shift-encoded (mDIXON Quant^®^) MRI sequence was used primarily for PDFF estimation, with T2* estimation as a secondary measure. This method has been previously validated against MR spectroscopy and histologic quantification as an accurate measure of PDFF across different scanner vendors [14]. A fast multi-echo 2D Gradient and Spin Echo (mGRASE) sequence with fat suppression was used for hepatic T2 estimation within an achievable breath-hold duration [15]. This sequence has demonstrated excellent agreement of T2 values with the established reference standard Carr-Purcell-Meiboom-Gill sequence [16, 17] and effectively minimizes influence of fat on T2 estimation [15].

## Methods

This prospective study was Institutional Review Board-approved (IRB ID 2023-0428) and was conducted in a HIPAA-compliant manner. Data were obtained on a single MRI scanner at a single institution. Written informed consent was obtained from participants aged 18 years and older. For participants less than 18 years of age, consent was obtained from parents/guardians and assent was obtained from participants aged 11 to 17 years.

### Study participants

Participants aged 8 to 30 years, with no MRI contraindications, who could remain still in an MRI scanner without sedation and who could hold their breath for at least 15 s were recruited for this study. The study aimed to include approximately one-third of participants with a prior diagnosis of hepatic steatosis, one-third of participants with other chronic liver diseases, and one-third of participants without known liver disease. Asymptomatic individuals without known liver disease were recruited through hospital-wide emails and advertisements. Patients with liver disease were identified and recruited using previous patient diagnoses listed in the Department of Radiology’s electronic medical records. Enrolled participants without known liver disease were reassigned to the hepatic steatosis subgroup if their study-measured liver PDFF was ≥ 6% [18].

### MRI acquisition

All participants were scanned on a 1.5T clinical MRI scanner (Ingenia, Philips Healthcare; Best, the Netherlands) using a torso coil with a combination of 16 anterior and 12 posterior elements embedded in the patient table. Respiratory bellows were placed at the mediastinum. The imaging protocol included a 3D six-echo mDIXON Quant^®^ acquisition for simultaneous PDFF and T2* mapping and a single axial-slice 2D nine-echo mGRASE acquisition with fat suppression for T2 mapping. Both mDIXON Quant and mGRASE sequences were performed twice with identical imaging parameters and spatial coverage: once during an end-expiration breath-hold (BH) and once free-breathing with respiratory gating (RG). During RG sequences, data was acquired only during the quiescent expiratory phase to minimize respiratory motion-induced artifacts. The mGRASE slice was prescribed at the widest part of the mid-liver in the transverse plane, and the central part of the transverse slab for 3D mDIXON Quant was positioned at the same slice location. Sequence parameters for the 6-echo CSE mDIXON Quant acquisition include FOV = 346 × 400 mm^2^ and 40 slices of 10 mm thickness, acquired in-plane resolution = 3 × 3 mm^2^, a sensitivity encoding (SENSE) acceleration factor (R) = 2, echo spacing = 0.7 ms, and TR/TE/flip angle of 5.3 ms/0.93 ms/5°. Sequence parameters for the single-slice 9-echo mGRASE acquisition include FOV = 343 × 400 mm^2^, acquired in-plane spatial resolution = 3 × 3 mm^2^, SENSE factor = 2, echo spacing = 7.4 ms, and TR/TE/flip angle = 1000 ms/15 ms/90°. The breath-hold time for mDIXON Quant acquisition was 7 s, and 14 s for the mGRASE acquisition. The predicted acquisition time was 15 s for RG mDIXON Quant and 25 s for RG mGRASE, based on a rate of 20 respiratory cycles per minute. The actual scan time for RG scans varied depending on the participants’ respiratory rates and were estimated using the physiology log files from all subjects. If significant motion induced artifacts were observed in either BH or RG sequences, the acquisition was repeated at the technologist’s discretion. The PDFF, T2*, and T2 maps were generated on the scanner using the vendor-provided, FDA-approved software.

### Image analysis

All MR images and scanner-generated PDFF, T2*, and T2 maps were anonymized and exported in DICOM format to external workstations. Two blinded pediatric radiologists (MK with 5 years of experience post-fellowship, JMK with 1 year experience as a pediatric radiology fellow) independently graded the BH and RG mDIXON Quant sequences for image quality, focusing on motion-induced artifacts and noise breakthroughs in the liver region. Images were graded as follows: 1 – poor, with severe artifacts, 2 – fair, with some artifacts but sufficient artifact-free area to draw ROI, and 3 – good, with no artifacts. In cases where acquisitions were repeated at the technologist’s discretion, acquisitions with the highest image quality were used for comparative analysis of BH and RG acquisitions.

**Fig. 1:**
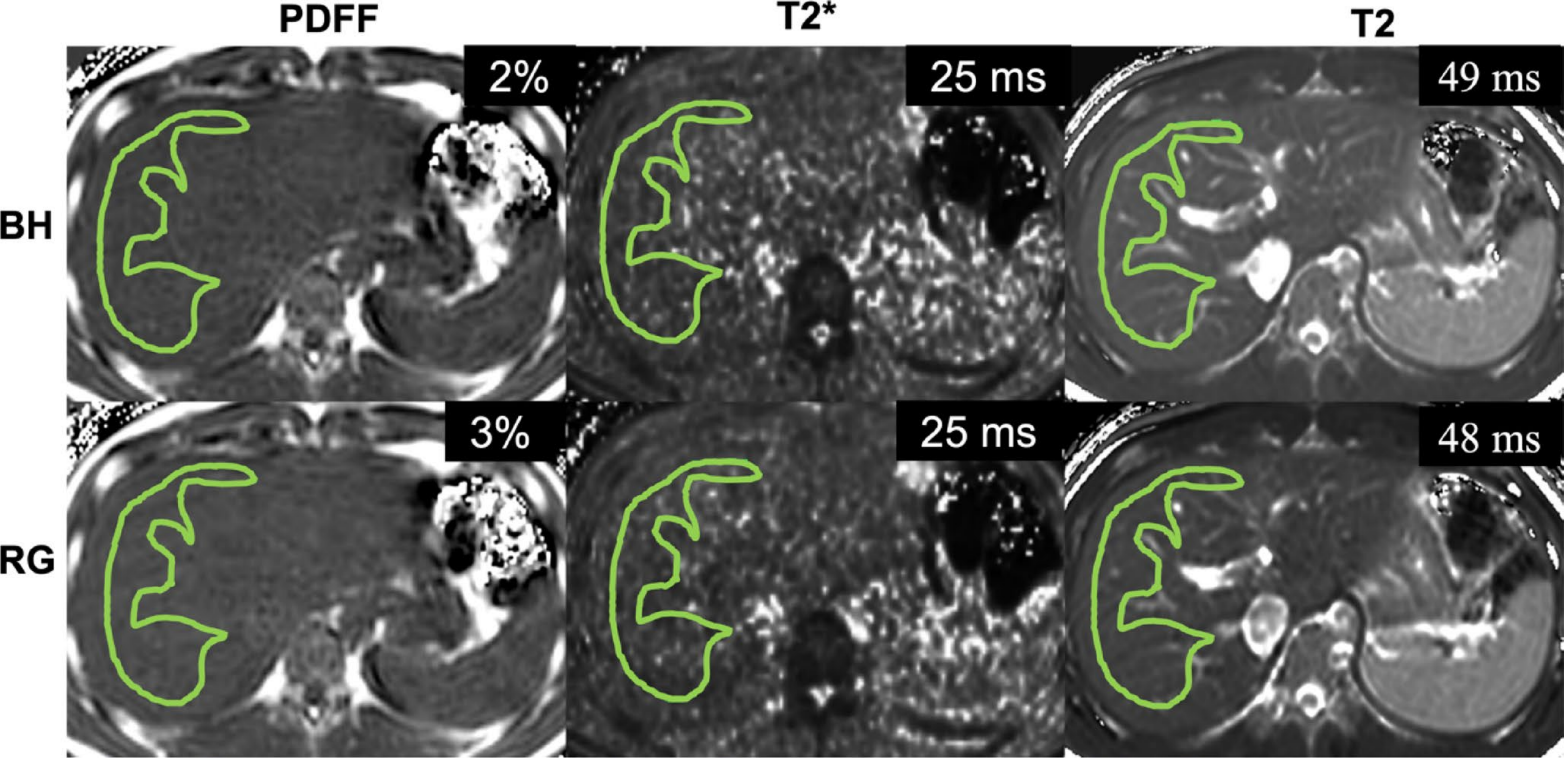
Representative PDFF, T2*, and T2 maps with ROIs and mean values for BH (top) and RG acquisitions (bottom) from a participant without liver disease (20-year-old male, BMI 24 kg/m^2^)

Manual regions of interest (ROI) were drawn independently by three observers: the same two radiologists (MK and JMK) using commercially-available post-processing software (IntelliSpace Portal v10.1; ISP, Philips Healthcare; Best, the Netherlands) and by an image analyst with 2 years of research experience (AKR) using in-house MATLAB software (The MathWorks™ Inc., Natick, MA, USA). Freehand ROIs with a minimum size of 200 mm^2^ were drawn on the right hepatic lobe while carefully avoiding any large visible artifacts, large blood vessels, bile ducts, and excluding the liver capsule (Fig. 1). ROIs were drawn in a mid-liver slice on PDFF and T2* maps that most closely matched the T2 map slice location. Additionally, upper and lower liver ROIs were drawn by both radiologists on PDFF and T2* maps to assess spatial homogeneity across liver regions. All ROIs were carefully drawn to approximately match the anatomic locations between BH and RG maps. The mean and standard deviation of the measured values from each ROI were recorded and used for further analysis. Comparative analysis between RG and BH acquisitions was conducted using the mean ROI values averaged across observers.

In a subset of participants where multiple RG mDIXON Quant or mGRASE acquisitions were obtained secondary to visible motion-induced artifacts, one pediatric radiologist (JMK) drew additional freehand ROIs on a mid-liver slice in the right hepatic lobe on images from both the initial acquisition and repeated acquisition used for comparative analysis to evaluate the impact of image quality on repeatability of the PDFF, T2* and T2 values.

### Statistical analysis

Normality was assessed using the Shapiro-Wilk test, and data were reported as either median and interquartile range or mean and standard deviation, as appropriate. Weighted kappa (κ) statistics were computed to assess the agreement of image quality grading between two clinical radiologist observers [19]. The κ was interpreted as follows: 0–0.20.20, poor; 0.21–0.40, fair; 0.41–0.60, moderate; 0.61–0.80, substantial; and 0.81–1.0.81.0, perfect agreement [20]. Inter-rater reliability of quantitative estimates between observers was assessed using intraclass correlation coefficients (ICC). Comparisons across liver ROIs from upper, mid, and lower regions were performed using ANOVA for normally distributed data and Kruskal-Wallis test for non-normally distributed data. Comparisons between BH and RG acquisitions were performed using independent samples t-tests for normally distributed data and the Mann–Whitney U test for non-normally distributed data, and agreement was assessed using intraclass correlation coefficients (ICC) and Bland-Altman analysis. Repeatability across the subset of scans repeated for motion was assessed using ICC. A p-value of < 0.05 was considered significant, and 95% confidence intervals were calculated as appropriate. ICC estimates was interpreted as follows: 0–0.19, very weak; 0.2–0.39, weak; 0.40–0.59, moderate; 0.60–0.79, strong; and 0.80–1.0, very strong [21]. Statistical analyses were performed using MATLAB (The MathWorks™ Inc., Natick, Massachusetts, USA) and GraphPad Prism (Dotmatrics, San Diego, California).

## Results

Twenty-one participants were recruited, with all but two successfully completing the MRI exam. One participant was unable to stay in the scanner for the duration of the exam, and another was excluded from further analysis due to significant artifacts and noise breakthroughs on both BH and RG mDIXON Quant images. Ultimately, 19 participants (9 males, mean age 15.4 ± 5.5 years [range: 9–28 years]) were included in the data analysis. The actual RG scan times measured from physiology log files were estimated to be 21 ± 6 s (range: 14–40 s) for mDIXON Quant and 28 ± 4 s (range: 24–40 s) for mGRASE acquisitions. On average, both RG acquisitions took about 14 s longer than corresponding BH acquisitions. The demographics and disease characteristics from baseline clinical diagnoses of the participants included in the analysis are summarized in Table 1. One participant enrolled without known history of liver disease was found to have a liver PDFF of 18% and was reassigned to hepatic steatosis subgroup.

**Table 1:** Demographics of the study sample

Number of participants	19
Age (year)	15.4 ± 5.5 (9–28)
Male-to-female ratio	9:10
BMI (kg/m^2^)	30.7 ± 11.0 (19.0–59.6.0.6)
Respiratory Rate (cycles/min)	20 ± 4 (9–29)
Clinical Indications	
No liver disease	6
Liver steatosis	9
Other liver disease	4

Radiologist agreement on subjective image quality (mDIXON Quant PDFF and T2*) was fair for BH (raw 60%, weighted κ = 0.31 [−0.29 – 0.91]) and RG (raw 75%, weighted κ = 0.40 [−0.30 – 1.10]) acquisitions. Image quality scores for BH acquisitions (median 2, IQR 1) were significantly higher (*p* < 0.01) than for RG acquisitions (median 2, IQR 0). There was no statistically significant difference between PDFF or T2* values of the upper, mid, and lower ROIs for either BH or RG maps (*p* > 0.5), and mid-liver values were used for all subsequent analysis. There was strong agreement among the measured PDFF, T2*, and T2 values from three independent observers (ICC = 0.91 to 0.99). The mean PDFF, T2*, and T2 values across the observers were used for further analysis.

**Fig. 2:**
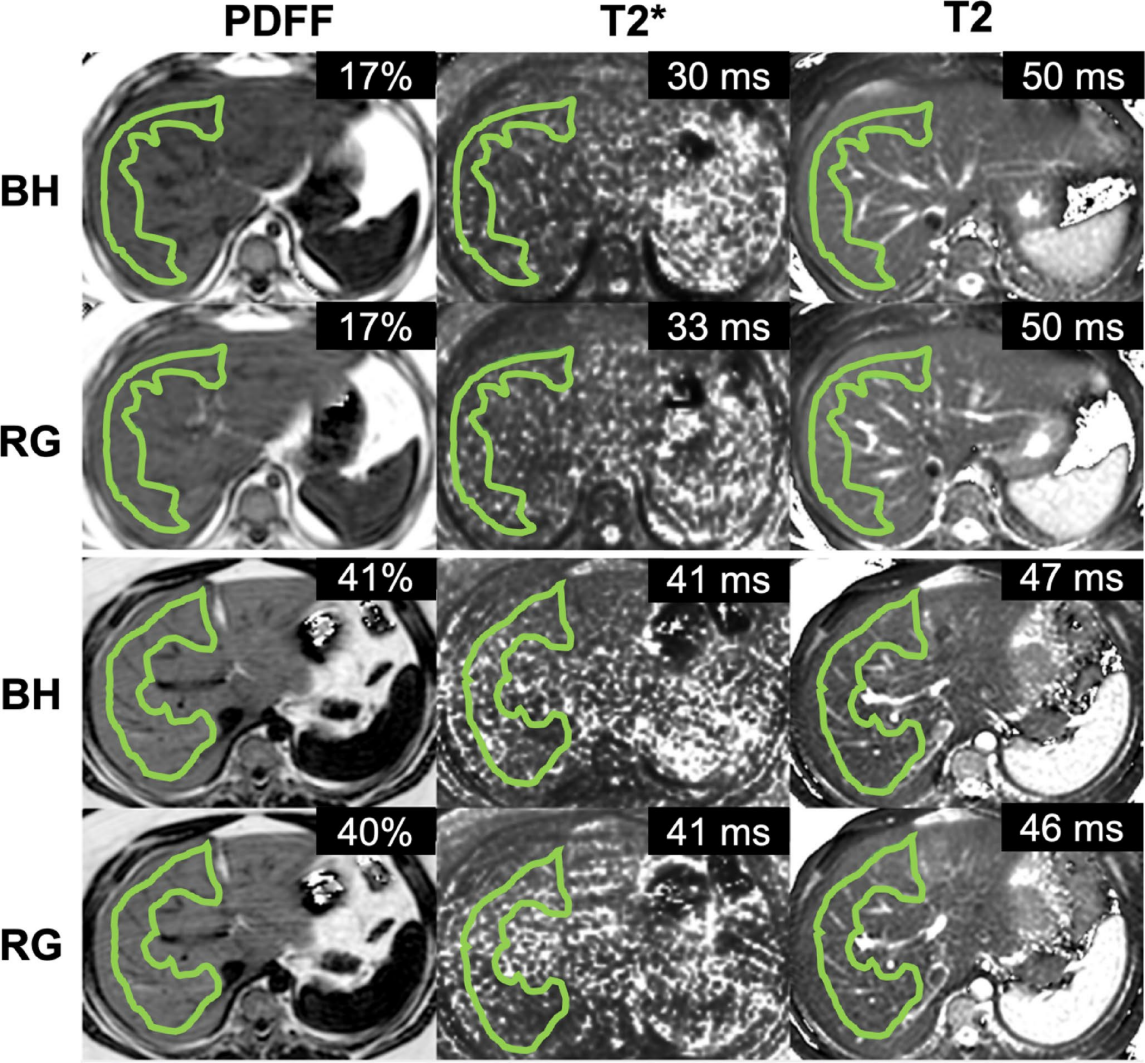
Representative PDFF, T2*, and T2 maps with ROIs and mean values for BH and RG acquisitions from a 9-year-old male (BMI 32 kg/m^2^) with MASLD and borderline MASH based on histology (top two rows) and a 19-year-old female (BMI 50 kg/m^2^) with MASLD (bottom two rows)

Hepatic PDFF values were non-normally distributed across participants with a median of 5% (IQR 3–32%) with a range of 1–43% for BH acquisitions, which were comparable (*p* = 0.69) to the PDFF values from the RG acquisitions (median 5%, IQR 3–32%, range: 2–43%). The mean hepatic T2* values from BH and RG mDIXON Quant acquisitions were normally distributed and had means of 27 ± 7 ms and 29 ± 8 ms, respectively with no significant differences (*p* = 0.51). The T2 values from mGRASE acquisitions were also normally distributed and comparable (*p* = 0.95) between BH (48 ± 8 ms) and RG acquisitions (49 ± 7 ms). Figure 2 presents scanner-generated PDFF, T2*, and T2 maps from BH and RG acquisitions of two representative participants with hepatic steatosis, with ROIs overlaid.

**Fig. 3:**
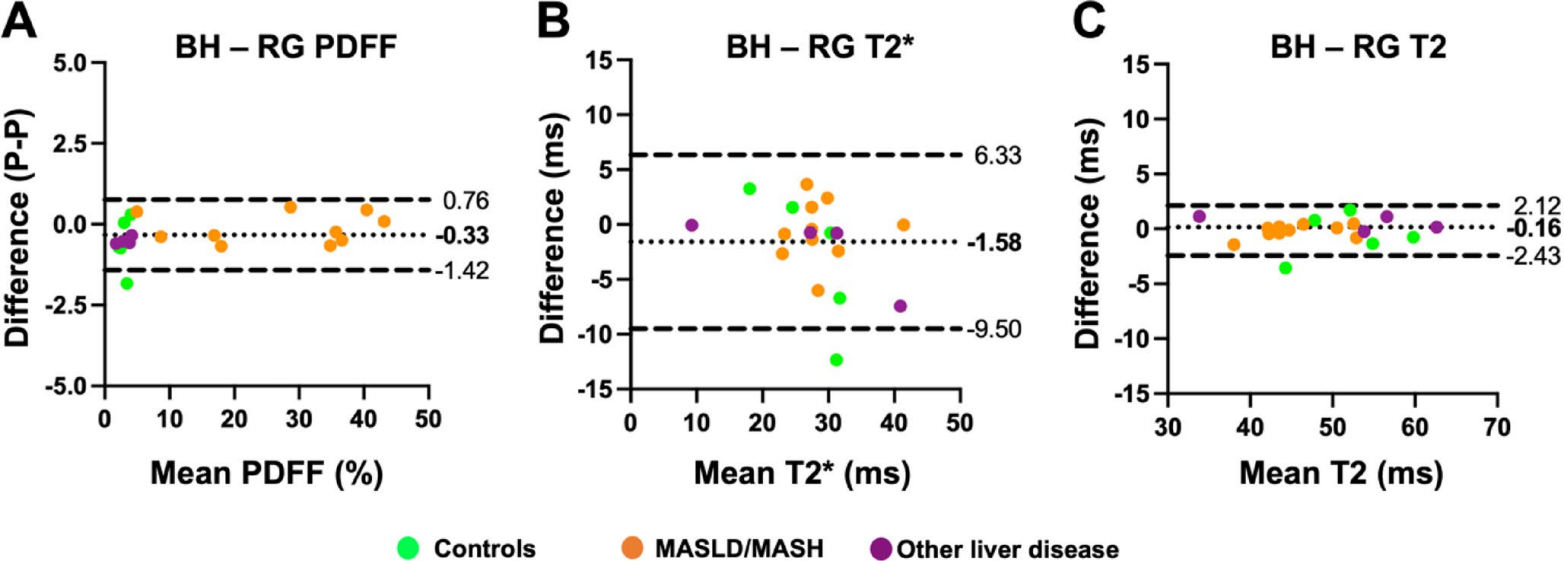
Bland Altman plots of **A** PDFF values, **B** T2* values, and **C** T2 values from BH and RG mDIXON Quant acquisitions

Figure 3A illustrates the Bland-Altman plots comparing PDFF values from BH and RG acquisitions. There was a very strong agreement (ICC = 0.99 [0.99–0.99]) with minimal bias (−0.33%, 95% Limits of Agreement: −1.42 to 0.76%) between the BH and RG measurements of hepatic PDFF. For T2* values, there was a very strong agreement (ICC = 0.85 [0.66–0.94]) between BH and RG acquisitions (Fig. 3B). The Bland Altman analysis showed a mean bias of −1.58 ms, with 95% Limits of Agreement of −9.50 to 6.33 ms between the measurements. There was a very strong agreement (ICC = 0.99 [0.97–0.99]) between T2 values obtained from BH and RG mGRASE acquisitions (Fig. 3C). Bland Altman analysis showed very little bias (−0.16 ms, 95% Limits of Agreement: −2.43 to 2.12 ms) between the measurements.

**Fig. 4:**
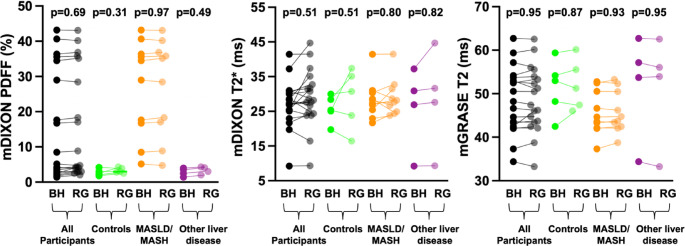
Line-connected scatter plots showing the mean PDFF, T2*, and T2 values obtained from BH and RG acquisitions for all participants, participants without liver disease, participants with hepatic steatosis, and participants with other liver diseases

The mean PDFF, T2*, and T2 values for all participants, as well as across participant groups for both BH and RG acquisitions are detailed in Table 2 and shown graphically in Fig. 4.

**Table 2:** Relaxometry values from BH and RG acquisitions

	PDFF (%)	T2* (ms)	T2 (ms)
All (*n* = 19)			
BH	5 [3–32] (1–43)	27 ± 7 (9–41)	48 ± 8 (34–63)
RG	5 [3–32] (2–43)	29 ± 8 (9–45)	49 ± 7 (33–63)
No liver disease (*n* = 5)			
BH	2 [2–3] (2–4)	26 ± 4 (20–30)	51 ± 6 (42–59)
RG	3 [3–4] (2–4)	29 ± 6 (16–37)	52 ± 5 (46–60)
Liver steatosis (*n* = 10)			
BH	32 [17–36] (5–43)	28 ± 5 (22–41)	46 ± 5 (37–53)
RG	32 [17–37] (5–43)	29 ± 5 (24–41)	46 ± 5 (39–53)
Other Liver Disease (*n* = 4)			
BH	3 [2–4] (1–4)	26 ± 12 (9–37)	52 ± 12 (34–63)
RG	4 [3–4] (2–4)	28 ± 15 (9–45)	51 ± 13 (33–63)

Data are presented as means and standard deviations or medians and IQR, with full ranges in parentheses

**Fig. 5:**
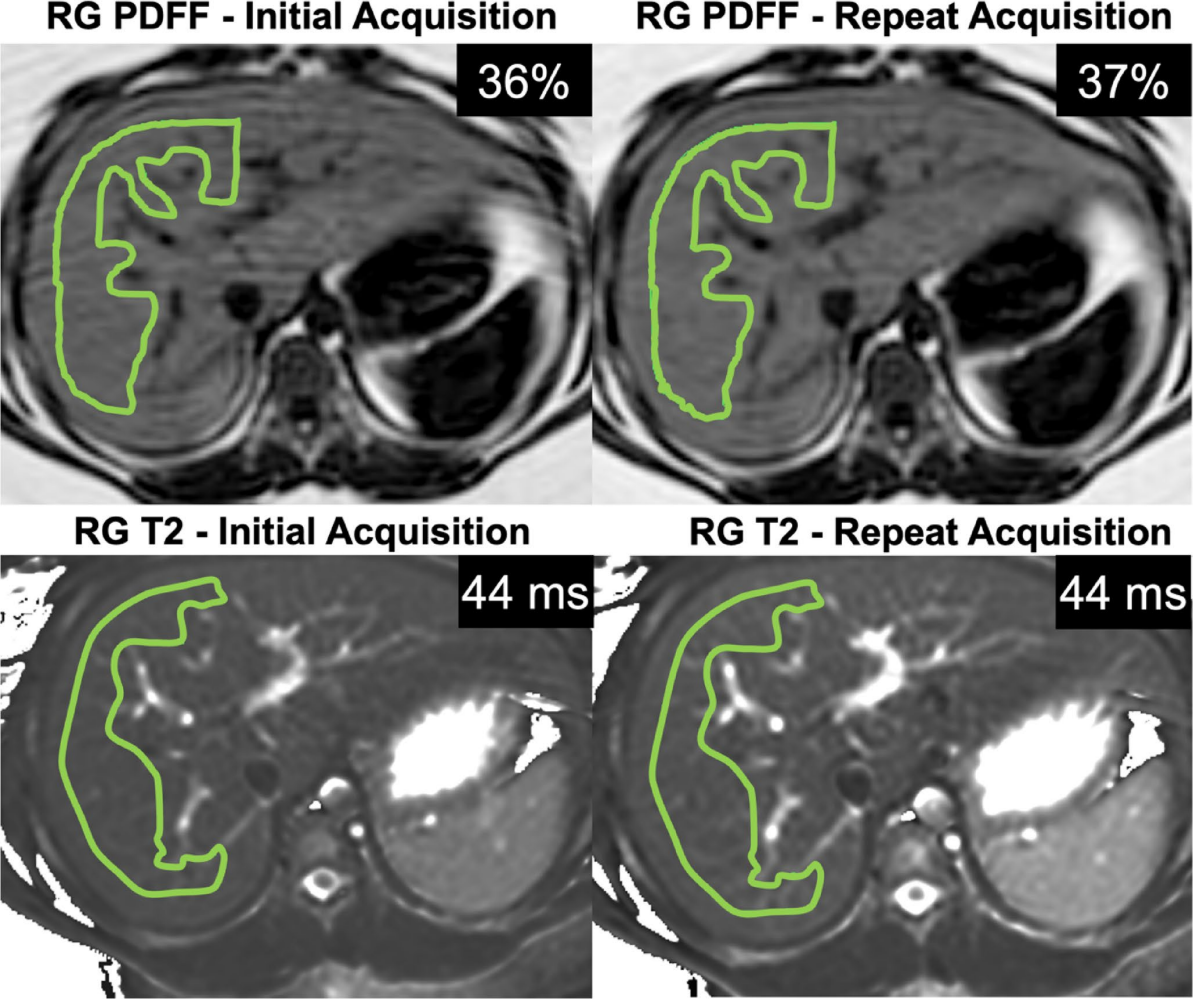
Representative PDFF, and T2 maps with ROIs and mean values for an initial RG acquisition with respiratory motion induced artifacts and for a repeat RG acquisition from a participant with MASLD and elevated liver stiffness (16-year-old female, BMI 37 kg/m^2^)

In 9 of 19 participants, the RG mGRASE acquisition was repeated at least once, and repeated values in this subset demonstrated strong agreement (ICC = 0.99 [0.98–0.99]) for T2 values. In 13 of 19 participants, the RG mDIXON Quant sequence was repeated at least once, and repeated values demonstrated strong agreement for PDFF (ICC = 0.99 [0.99–0.99]) and moderate agreement for T2* (ICC = 0.78 [0.42–0.93]) values. Representative images with ROIs are shown in Fig. 5. Although not analyzed for repeatability, the BH mGRASE acquisition was repeated at least once in 4 participants while the BH mDIXON Quant sequence was repeated at least once in 2 participants.

## Discussion

Our study compared PDFF, T2*, and T2 estimates obtained by BH and RG in children and young adults without and with diffuse liver diseases. Our results demonstrated that hepatic PDFF, T2*, and T2 values obtained from RG acquisitions were statistically comparable to those obtained from BH acquisitions. RG scan times were all under one minute, with median scan times under 30 s for both mDIXON Quant and mGRASE acquisitions with identical spatial resolution and coverage as the corresponding BH acquisitions. Additionally, hepatic PDFF and T2 measurements were consistent across RG acquisitions when acquisitions were repeated due to perceived respiratory motion artifacts. These results demonstrate that robust quantitative hepatic measurements can be obtained from an RG acquisition and do not necessarily need to be repeated, even if qualitative motion artifacts are observed during patient scanning.

The ability to obtain quantitative hepatic measurements without the need for breath-holding is a considerable advantage in clinical settings. This is especially relevant for patients who are unable to hold their breath due to age, respiratory conditions, or the need for sedation. The results of our study align with those of Motosugi et al., who demonstrated equivalent PDFF values between BH and RG acquisitions performed either with respiratory bellows or navigator echoes and limited liver coverage in adults [22]. While Motosugi et al. found no significant difference in image quality scores across BH, RG with navigator-echo, and RG with respiratory bellows acquisitions, more patients in this study had poor image quality on the free breathing acquisitions. As postulated by Motosgui et al., image quality on free breathing acquisitions may be degraded by irregular respiratory cycles or a high respiratory rate secondary to inappropriate respiratory gating.

Our findings are also similar to those from Gilligan et al. who compared hepatic PDFF and R2* measurements between a longer, 25–28 s BH and navigator-gated mDIXON Quant acquisitions in pediatric patients covering the whole liver [13]. Their results showed no significant difference and strong correlation between R2* measurements across the two sequences. Similarly, almost perfect concordance, although a statistically significant difference, was seen between the PDFF measurements. However, image quality was limited by motion-induced artifacts on 88.1% of scans due to the navigator echoes, which excite the hepatic parenchyma and make saturation effects possible. In contrast, respiratory bellows are used in our study which eliminates this possibility.

Image quality for all RG scans in our study was adequate, although image quality scores for BH scans were significantly higher compared to RG scans. These findings are again similar to those of Gilligan et al., who found significant differences in qualitatively graded motion-induced artifacts between BH and navigator-echo sequences, with navigator-echo sequences demonstrating clinically limiting motion-induced artifacts [13]. Among participants in the prior study by Gilligan et al., 40.4% had clinically limiting motion-induced artifacts on navigator-echo sequences without clinically limiting motion-induced artifacts on BH sequences. Despite this, no significant differences in PDFF measurements were observed in participants with substantial motion-induced artifacts between the BH and navigator-echo acquisitions, suggesting that PDFF measurements may be somewhat robust to motion artifacts. The worse image quality scores among free-breathing acquisitions when compared to breath-hold acquisitions in our study, as well as in the previous pediatric study by Gilligan et al. could be explained by the higher and irregular respiratory rates of pediatric subjects. Additional pediatric studies, including studies of even younger patient groups, are needed to understand the impact of age and respiratory rate on image quality and quantitative accuracy.

Spatial variation of PDFF and T2* values across liver regions was very small, in line with previous findings from de Padua V Alves V et al. [23]. Excellent inter-rater reliability was observed for all measured hepatic quantitative values from both RG and BH acquisitions across three independent raters with varying degrees of clinical experience. The biases observed between the two methods were of minimal clinical relevance, with − 0.33% for PDFF, −1.58 ms for T2*, and 0.16 ms for T2. These minimal biases, coupled with strong intraclass correlation coefficients (ICC = 0.99, 0.85, and 0.99 for PDFF, T2*, and T2) between the two acquisition techniques indicate that the RG technique is a reliable alternative to BH acquisitions.

While our study provides robust evidence supporting the use of RG acquisitions of quantitative images of the liver, it had a small sample size, narrow age range of participants, and limited spectrum of liver diseases, which may affect the generalizability of the findings. Further studies are needed to address these limitations. Future studies should evaluate the applicability of these findings in a larger cohort consisting of diverse liver diseases with a wide range of disease severity and across a broader age range. Future refinement of these respiratory-synchronized acquisition methods may be warranted to enhance their clinical utility.

In conclusion, free-breathing RG acquisitions offer a viable and reliable alternative to breath-hold acquisitions for estimating hepatic PDFF, T2*, and T2 values. This respiratory synchronized data acquisition approach holds promise of providing diagnostically accurate estimates of hepatic PDFF, T2*, and T2 while affording patient comfort, particularly in populations with limited breath-holding capabilities.

## Data Availability

No datasets were generated or analysed during the current study.
